# Cost-Effective Fabrication of Transparent Strain Sensors via Micro-Scale 3D Printing and Imprinting

**DOI:** 10.3390/nano12010120

**Published:** 2021-12-30

**Authors:** Rui Wang, Xiaoyang Zhu, Luanfa Sun, Shuai Shang, Hongke Li, Wensong Ge, Hongbo Lan

**Affiliations:** 1 Key Lab of Industrial Fluid Energy Conservation and Pollution Control, Ministry of Education, Qingdao 266520, China; wr1970627@163.com (R.W.); sunluanfa@163.com (L.S.); shangshuai0602@163.com (S.S.); LHK1164072308@163.com (H.L.); g17864208754@126.com (W.G.); 2 Shandong Engineering Research Center for Additive Manufacturing, Qingdao University of Technology, Qingdao 266520, China

**Keywords:** multiwalled carbon nanotube, strain sensors, transparent sensors, micro-scale 3D printing

## Abstract

The development of strain sensors with high sensitivity and stretchability is essential for health monitoring, electronic skin, wearable devices, and human-computer interactions. However, sensors that combine high sensitivity and ultra-wide detection generally require complex preparation processes. Here, a novel flexible strain sensor with high sensitivity and transparency was proposed by filling a multiwalled carbon nanotube (MWCNT) solution into polydimethylsiloxane (PDMS) channel films fabricated via an electric field-driven (EFD) 3D printing and molding hybrid process. The fabricated flexible strain sensor with embedded MWCNT networks had superior gauge factors of 90, 285, and 1500 at strains of 6.6%, 14%, and 20%, respectively. In addition, the flexible strain sensors with an optical transparency of 84% offered good stability and durability with no significant change in resistance after 8000 stretch-release cycles. Finally, the fabricated flexible strain sensors with embedded MWCNT networks showed good practical performance and could be attached to the skin to monitor various human movements such as wrist flexion, finger flexion, neck flexion, blinking activity, food swallowing, and facial expression recognition. These are good application strategies for wearable devices and health monitoring.

## 1. Introduction

Flexible strain sensors play an important role in applications such as health monitoring [1,2,3], electronic skin [4,5,6], wearable devices [7,8,9,10,11,12], and human-computer interactions [13]. Piezoresistive strain sensors have played an important role in the field of flexible strain sensors due to their simple reading mechanism. In which, Sensitivity and tensile properties are two important parameters for evaluating the performance of sensors [14,15]. Parameters such as response time and dynamic stability are also crucial for their practical applications. Hence, the development of flexible strain sensors with high sensitivity and stretchability is crucial for monitoring of human motion. However, most strain sensors are opaque to ensure sensitivity and tensile properties, which limits their practical application in areas such as the face, neck, and other visible areas.

Here, polydimethylsiloxane (PDMS) [16,17,18], thermoplastic polyurethane (TPU) [19], Ecoflex [20], and natural rubber [21] were selected as base materials for flexible strain sensors. PDMS is optically transparent, flexible, scalable, and can be easily combined with electronic materials; thus, it is a popular material for flexible wearable strain sensors. Different conductive fillers such as metallic nanoparticles [22,23], silver nanowires (AgNWs) [24,25], carbon nanotubes (CNTs) [26,27,28], and graphene [29,30] have been used to prepare strain sensors. Of these, metal nanoparticles and AgNWs have high conductivity and can obtain high conductivity with low filler amounts. However, metal fillers are difficult to process, and AgNWs are rarely used to fabricate flexible strain sensors due to their costs [31,32]. Although graphene has a high carrier mobility and large specific surface area, it is difficult to uniformly disperse it in polymers, which limits the application of graphene in flexible strain sensors [33]. CNTs have a small percolation threshold due to its nanometer scale, high aspect ratio, and excellent electrical conductivity. CNTs have stable properties and maintain their original electrical properties even under tension and bending, making them particularly suitable for the preparation of flexible strain sensors [34,35]. The combination of PDMS and CNTs has been used to prepare strain sensors with high sensitivity and stretchability. However, published CNTs are mostly randomly distributed in the polymer matrix, thus resulting in compromised optical transparency of the strain sensors.

Recent research has been devoted to modifying the conductive structure of nanomaterials to enhance their sensor performance, including microcracks, microspheres, and microporosity. Of these, microcrack structures inspired by the spider structure have been widely used to prepare various flexible strain sensors with high sensitivity. For example, Zhou et al. [36] sprayed CNT on TPU and produced cracks by pre-stretching. The prepared CNT/TPU strain sensor has an ultra-wide detection range of up to 300% with a gauge factor of 428.5 in the strain range of 0–100%, 9268.8 in the strain range of 100–220%, and 83,982.8 in the strain range of 220–300%; these metrics satisfied the demands for monitoring various fine and large deformations. By combining the cracking mechanism and tunneling effect, Chen et al. [37] designed a sensor with an ultra-low detection limit of 0.01 and an ultra-wide detection range (>100%)—it also had an excellent/fast response and good repeatability. With these properties, sensors can be used to detect subtle physiological activities (pulses, coughing, and swallowing) as well as walking, leg lifting, and squatting. However, these reported methods require time-consuming procedures and wastage of materials, limiting the commercialization of flexible strain sensors.

3D printing is an advanced manufacturing strategy widely used in electronics, biomedicine, and aerospace. If offers low processing costs, high manufacturing accuracy, and excellent productivity. Research has been devoted to the application of 3D printing technology to flexible strain sensor manufacturing [38]. 3D printing technology offers a promising approach to fabricate flexible strain sensors compared with conventional manufacturing approaches. Currently, processing methods based on fused deposition modeling (FDM) and direct ink writing (DIW) are widely used to build strain sensors. Christ et al. [39] fabricated bidirectional strain sensors using a nozzle to print TPU as a substrate and another nozzle to print a mixture of TPU and MWCNT as a conductive filler. Wajahat et al. [40] prepared flexible strain sensors via a CNT ink formulated from multiwall nanotubes (MWNTs) and polyvinylpyrrolidone (PVP). The sensors exhibited high stability with a gauge factor of 13.07. These could be applied to human motion monitoring and remote monitoring of robotic devices. However, the raw material used in FDM technologies must be pre-processed under high temperature before printing, and the surface of the printed component is rough. The DIW 3D printing approach can easily clog the nozzle, and the component requires post-processing.

In this study, an efficient, low-cost, and mass-produced EFD micro-scale 3D printing [41,42,43,44,45,46] and molding hybrid method is proposed to fabricate PDMS channel films. MWCNTs were filled into PDMS films with channel networks to prepare flexible strain sensors with high sensitivity, transparency, and stretchability. Three different ratios of PDMS prepolymer and cross-linker (10:1, 15:1, and 20:1) were chosen to test the sensitivity and stretchability of the strain sensor, and the optimal ratio was chosen to investigate the detection range, sensitivity, stability, transparency, and responsiveness of the sensor. The final fabricated flexible strain sensors with up to 84% transparency exhibited ultra-high sensitivity (gauge factor up to 1500 at 20% tensile strain) and over 8000 stretch-release cycles. The feasibility and effectiveness of the sensor were demonstrated by mounting the sensor on various parts of the human body for testing. The proposed EFD micro-scale 3D printing method opens up new prospects for the fabrication of flexible transparent strain sensors for practical applications in various fields.

## 2. Materials and Methods

### 2.1. Materials

The MWCNT dispersion (XFWPM-H-M31) was purchased from XFNANO Materials Tech Co., Ltd., Nanjing, China, and dispersed in water. The concentration of the MWCNT was 14 wt.%. The length of the CNT was approximately 10 μm, and the outside diameter was greater than 50 nm. The PDMS prepolymer and cross-linker (Sylgard 184) were purchased from Dow Corning, Midland, MI, USA. Conductive silver ink (CON-EHD50) purchased from BroadTeko, Beijing, China. CON-EHD50 is a nano-silver conductive ink custom developed for electrohydraulic inkjet printing (EHD jetting, also known as near-field electrospinning).

### 2.2. Fabrication of the Flexible PDMS Film with Channel Structures

**Figure 1:**
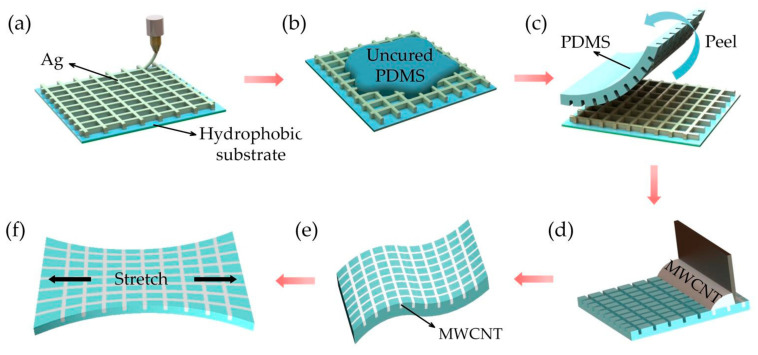
Fabrication process of the flexible strain sensor with the embedded MWCNT network.

A 20-µm-thick layer of liquid PDMS (prepolymer to cross-linker ratio of 10:1; uniformly mixed and vacuumed) was spin-coated on the glass surface and cured for 30 min to form a hydrophobic surface. Polymer printing generally adopts FDM technology, which requires pre-treatment at high temperature. The intersection points cannot be lapped when printing the grid structure, which is unfavorable for PDMS films with manufactured channel structures [41]. Silver grid printing has good consistency and neat intersections and is a simple process, which is suitable for master molds of PDMS films with channel structure manufacturing [42]. The silver grid structure (width of 20 µm; five layers) was deposited on the hydrophobic glass surface using EFD microscale 3D printing technology and cured at 100 °C for 30 min to enhance the hardness of the silver paste structure (Figure 1a). The liquid PDMS prepolymer and cross-linker were uniformly mixed at a ratio of 15:1 (10:1 or 20:1) and degassed for 30 min under vacuum. Afterward, the mixture was drop cast onto PDMS films with a metallic silver grid and then cured on a hot plate at 100 °C for 30 min (Figure 1b). The PDMS film with channel structures was peeled off from the PDMS substrates after curing to yield a PDMS channel structure film with flexibility and transparency (Figure 1c).

### 2.3. Fabrication of the Transparent Strain Sensor with an Embedded MWCNT Network

The MWCNT was chosen as the sensing material due to good electrical properties and chemical stability [47,48]. Figure S1 shows the TGDSC curves of MWCNTs. There is a strong heat absorption peak on the DSC curve at around 100 °C, which is the evaporation of heavy adsorbed water of the sample. There is a more obvious weight loss on the corresponding TG curve, indicating the complete evaporation of water from MWCNTs after 7 min of drying at 100 °C. In the range of 100–360 °C, the TG curve tends to be stable, and after exceeding 360 °C, there is a weight loss on the TG curve, indicating that there is no significant change in the sample weight of MWCNT in the range of 100–360 °C, and the conductivity of MWCNT is not affected in this temperature range. Due to the chosen MWCNT solution is aqueous, and PDMS is hydrophobic; thus, the surface of the PDMS films was hydrophilically treated with vinyltriethoxysilane solution (1% vinyltriethoxysilane dispersed in water and soaked for 2 h). The MWCNT solution was then poured on the side surface of the PDMS and dragged at a constant speed with a blade. During dragging, the MWCNT solution was filled into the PDMS channel due to the capillary forces (Figure 1d). The MWCNT in the channel was dried on a hot plate at 70 °C for 3 min, and the excess MWCNT was removed from the PDMS membrane surface by ethanol-assisted scraping. The embedded MWCNT-conducting network was fabricated inside the PDMS channels (Figure 1e). To obtain a reliable electrical connection, a thin layer of silver ink was first spread on the two ends of the conductive route, followed by curing at 70 °C for 1 h to decrease the contact resistance. Later, a thin and flexible conductive tape was pasted on the silver ink, and there was a thin layer of liquid metal between the conductive tape and the silver ink, resulting in a reliable electrical connection. Finally, the contact points were encapsulated by the PDMS, thereby providing protection for reliable operation.

### 2.4. Measurement and Characterizations

To test the strain sensor performance of the embedded MWCNT network, the sensor film was stretched to different strains using a tensile tester, and the resistance changes were measured using a resistance tester (Applent, Edison, NJ, USA, AT516). A UV-Vis spectrophotometer (METASH, Shanghai, China, UV-6100) was used to measure the optical transmittance of the sensor. The structural morphology of the channel crack structure was characterized using a field emission scanning electron microscope (SEM) (SU8010, HITACHI, Tokyo, Japan).

## 3. Results and Discussion

### 3.1. Performance Characterizations of the Transparent Strain Sensor with the Embedded MWCNT Network

**Figure 2:**
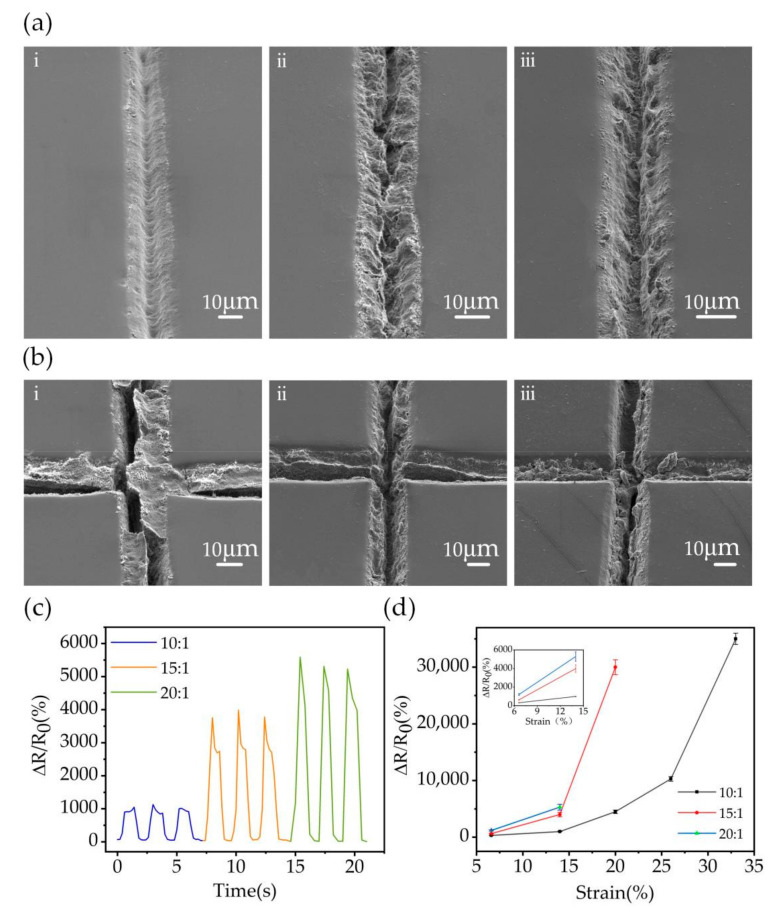
Performance of sensors prepared with different ratios of PDMS prepolymer and cross-linker. (**a**) MWCNT filling effect of PDMS films prepared with different ratios: 10:1, 15:1, and 20:1. (**b**) Stretching effect after filling MWCNT with PDMS films of different proportions. (**c**) Variation of ΔR/R_0_ at 14% tensile strain for sensors prepared with different PDMS ratios. (**d**) Maximum tensile strain of the sensors prepared with different PDMS ratios.

The width of the non-transparent channel was set at 20 μm to obtain relatively good conductivity and proper transparency of the strain sensor. For printing metallic silver meshes, a width of 20 µm is more suitable and easier to print. Meanwhile, the size of the channel spacing was kept to 1000 μm. When the channel width was constant, a smaller channel spacing led to poor transparency. Therefore, a spacing of 1000 μm was selected to fabricate the sensor to ensure good transparency. Meanwhile, the PDMS prepolymer and cross-linker ratios directly affect the tensile performance and sensitivity of the strain sensors and also impact the conductivity of the sensor. Therefore, experiments to test the different ratios of PDMS prepolymer and cross-linker were performed (Figure 2). The ratio of PDMS prepolymer and cross-linking agent commonly used in general experiments is 10:1. Figure 2a(i–iii) shows the filling effect of MWCNT with PDMS prepolymer and crosslinker ratios of 10:1, 15:1, and 20:1, respectively. The results show that MWCNT is well-filled at the 10:1 ratio with no obvious cracks in the channel; the MWCNT exhibits few cracks at the 15:1 ratio, and the MWCNT is less filled at the 20:1 ratio with more obvious cracks in the channel. The ratio of PDMS has a strong influence on the interfacial stability of the conductive structures; the stability of the channel network improves as the PDMS ratio increases, thus effectively filling the MWCNT into the channel as the blade cuts across the channel surface. The PDMS becomes soft when the PDMS ratio is too large, causing the channel to close when the blade cuts across the channel. The MWCNT crack expansion in the channel under the same stretching condition for different PDMS ratios is shown in Figure 2b. It is obvious that the MWCNT crack expansion is smaller at the 10:1 ratio (Figure 2b(i)), and the MWCNT crack expansion is the largest at the 20:1 ratio(Figure 2b(iii)). On the one hand, the filling amount of MWCNT has an effect on the crack extension during stretching. Figure S2 shows the dispersion of MWCNT in the matrix and the microscopic morphology of MWCNT filling in the PDMS channel. It can be seen that MWCNT is well dispersed in the matrix, MWCNT has a high aspect ratio, and the fibrous MWCNT can effectively form a bridge structure to support the connection between cracks when stretched. The more MWCNT filling, the slower the crack formation when stretched, which enables the sensor to withstand greater tensile strain. On the other hand, the PDMS channels have a protective effect on the MWCNT structure, and PDMS with increasing ratio of prepolymer and cross-linker becomes softer, leading to a decrease in the protective effect of the channels on the MWCNT structure.

The mechanism of flexible strain sensor with embedded MWCNT network is mainly derived from crack propagation. Tensile strain changes the crack structure in the channel. The reconnection or disconnection of the crack structure fundamentally changes the conductivity of the strain sensor. Thus, the sensitivity of the sensor becomes larger with increasing ratio of PDMS prepolymer to cross-linker. The relative resistance changes of sensors prepared with different proportions of PDMS prepolymer and cross-linking agent under a tensile strain of 14% are shown in Figure 2c. The normalized resistance (ΔR/R_0_) is defined as the ratio between the resistance change (ΔR) to the initial resistance (R_0_), where ΔR = R − R_0_.

It can be seen that the change in normalized resistance gradually becomes larger as the PDMS prepolymer to cross-linker ratio increases. The normalized resistance value is 1000, below a ratio of 10:1. The ΔR/R_0_ increases to 4000 when the ratio increased to 15:1, and the maximum value is 5300 when the ratio is 20:1. The high sensitivity of the sensor is achieved by the disconnection and reconnection of the MWCNT structure. At the same tensile strain, the filling amount of MWCNT with a 10:1 ratio of PDMS prepolymer to cross-linker is significantly better than the filling amount of MWCNT with a 15:1 and 20:1 ratio of PDMS prepolymer to cross-linker. Therefore, the fibrous MWCNT can achieve bridge lap during the tensile process, resulting in insignificant changes in the sensor resistance value, but the stretching range of the sensor gets improved. Figure 2d shows the maximum tensile strain that the PDMS can withstand at different ratios. The results show that the sensor prepared with a PDMS ratio of 20:1 can only withstand 14% of the tensile strain, while the sensor prepared with a PDMS ratio of 10:1 can withstand a maximum strain of 33% (20% at 15:1). The PDMS ratio impacts both the resistance change and the tensile performance of the sensor. Although the sensor prepared with a PDMS ratio of 20:1 has high sensitivity, it can only withstand a maximum of 14% tensile strain. Due to the protective effect of PDMS channels on MWCNT, when the ratio of PDMS prepolymer and cross-linker is 10:1, the MWCNT cracks in PDMS channels are more stable, the fiber structure of MWCNT can form a bridge structure within 30% of the tensile strain. When the ratio of PDMS prepolymer and cross-linker was 20:1, the PDMS channel network became soft, and the protective effect of PDMS channel network on MWCNT was reduced during stretching, resulting in the fiber structure of MWCNT being destroyed when the tensile strain exceeded 14%. Therefore, the dynamic stability is poor at 14% strain, and the sensor can only be applied to the motion monitoring of the human body with a strain below 14%. The sensitivity of the sensor prepared with a PDMS ratio of 10:1 is relatively low—it has a tensile strain of 30% and is used for the human motion monitoring of a strain below 30%. The sensor prepared with a PDMS ratio of 15:1 has good sensitivity and can monitor human motion with a strain below 20%. The ratio of PDMS prepolymer and cross-linker has a dual influence on the sensitivity and detection range; thus, a comprehensive consideration should be made for practical applications.

**Figure 3:**
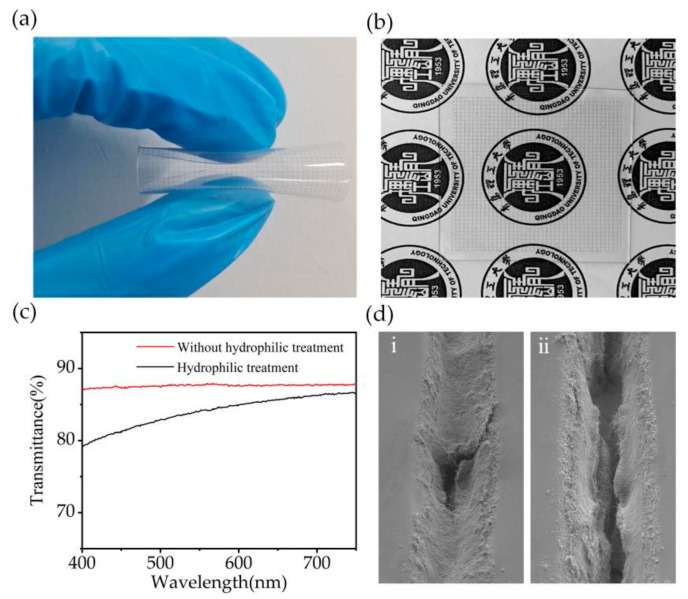
(**a**) Snapshot of the strain sensor after bending. (**b**) Snapshot of the strain sensor located on the logo of Qingdao University of Technology. (**c**) Light transmission rate of strain sensors without hydrophilic treatment and with hydrophilic treatment. (**d**) MWCNT filling effect of hydrophilic-treated PDMS films and non-hydrophilic-treated PDMS films.

Considering the tensile strain and sensitivity performance of the sensor, a 15:1 ratio of PDMS mixture was used to prepare the flexible strain sensor with the embedded MWCNT network. Figure 3a,b shows the macroscopic images of the fabricated sensor in different states, respectively, illustrating its good flexibility and transparency.

The PDMS films were subjected a hydrophilic treatment to obtain a better filling effect of MWCNT. After the hydrophilic treatment, a thin layer of milky substance appeared on the surface of the PDMS film, which impacted the optical transmittance of the sensor. Therefore, we tested the optical transmittance of hydrophilic-treated and non-hydrophilic-treated sensors separately (Figure 3c). The transmittance of the sensor without hydrophilic treatment was 87%, which was slightly reduced to 84% after hydrophilic treatment. The MWCNT filling effect of the PDMS channel film without hydrophilic treatment is poor and requires multiple scrapings (Figure 3d(ii)). Whereas the filling rate of MWCNT of the hydrophilic-treated PDMS film was significantly increased (Figure 3d(i)), which improves the conductivity of the sensor. Therefore, the preparation of PDMS films subjected to hydrophilic treatment with flexible strain sensor with an embedded MWCNT network can guarantee good conductivity and appropriate light transmittance.

**Figure 4:**
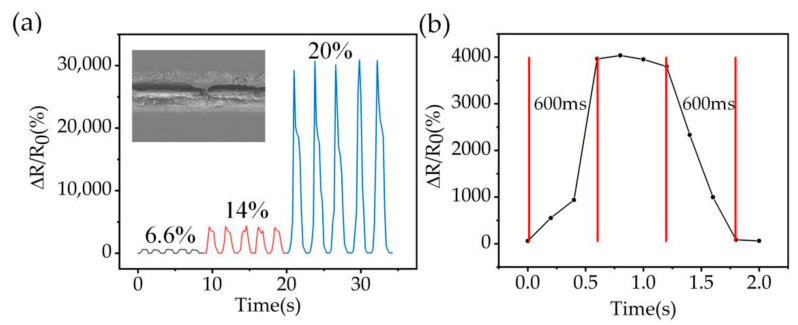
Sensor performance at PDMS ratio of 15:1. (**a**) Normalized resistance of the strain sensor at 6.6%, 14%, and 20% strain. The inset shows the SEM of crack extension after stretching MWCNT (**b**) Response/recovery time of the sensor at a strain of 14%.

To obtain better detection sensitivity, and be able to apply the sensor to multiple parts of the human body for monitoring. A PDMS prepolymer and cross-linker ratio of 15:1 was selected to prepare the flexible strain sensor with embedded MWCNT networks; the channel width was 20 μm, depth was 30 μm, and channel spacing was 1000 μm. The performance of the strain sensor is shown in Figure 4. The gauge factor (GF) is an important parameter to evaluate the performance of the sensor and defines the sensitivity of the sensor. Figure 4a illustrates the typical response of the strain sensor to different strains. The GF values of the strain sensors ranges from 10 to 1500 when the strain increased from 6.6% to 20%; the GF of the strain sensor reaches 285 at 14% strain, which are comparable to those of the MWCNT based strain sensors in most reported work [2,9,10,12,28,48] (Table 1), and the sensors manufactured in this work have better mechanical stability than other sensors with transparency. The cracking of the strain sensor is maximized when the strain exceeds 20% resulting in fracture of the MWCNT structure, and the resistance reaches zero. Meanwhile, the normalized resistance variation is unstable in the 20% strain range. Then, to conduct a more in-depth study on the performance of the sensor, the response time of the sensor was tested. Figure 4b shows that the sensor was subjected to a stretch-release cycle at 14% strain, and the strain was applied at 4 mm s^−1^ and held for 8 s before being released. The experimental results show that the manufactured strain sensors have a response time and recovery time of 600 ms, indicating a good response and recovery time, which has good potential for applications in human motion detection.

**Figure 5:**
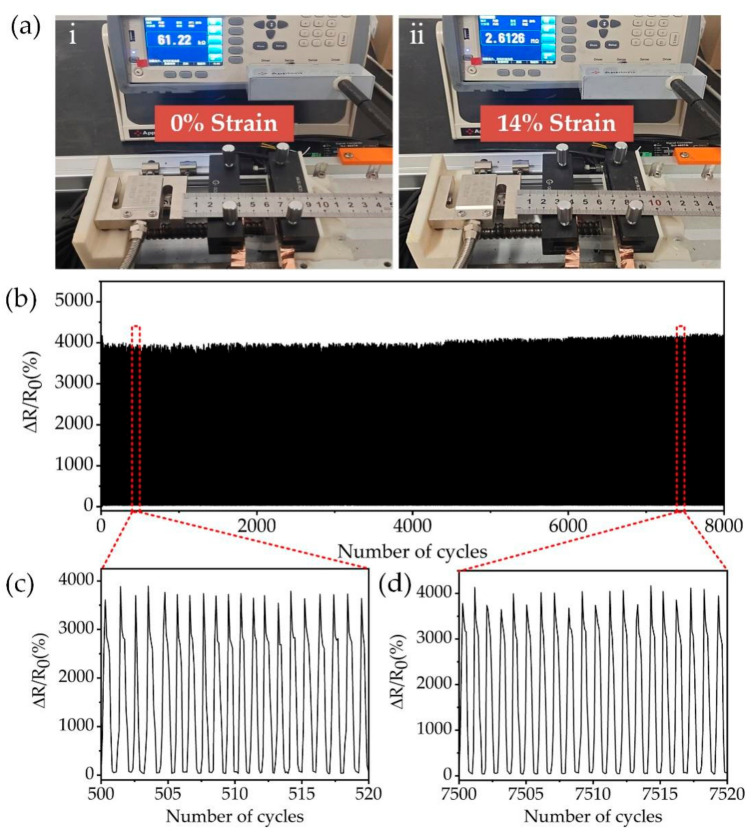
Stability performance of the strain sensor with the embedded MWCNT network. (**a**) Resistance changes of the sensor during 10,000 stretch-release cycles. (**b**) Resistance changes between 500 and 520 stretch-release cycles. (**c**) Resistance changes between 7500 and 7520 stretch-release cycles. (**d**) Resistance changes at 0% and 14% strain.

The mechanical stability of strain sensors is another important parameter for evaluating the performance of strain sensors. In practical applications, the sensor needs to undergo thousands of stretch-release processes to maintain almost constant resistance. The mechanical stability of the flexible strain sensor with the embedded MWCNT network was investigated using a homemade tensile test platform. The stretch-release process of the sensor is shown in the Figure 5a. The sensor is fixed on a tensile tester, and the resistance change of the sensor in the range of 0–14% of tensile strain is measured using a resistance tester, and the measured data is transmitted to the PC for processing. Figure 5a(i,ii) shows the resistance values of the sensor measured at 0% and 14% of the tensile strain, respectively. Figure 5b shows the relative resistance changes of the sensor under stretch-release cycles. The sensing stability was tested continuously for 8000 stretch-release cycles at 14% strain. The ΔR/R_0_ always varies from 0% to 4200%, while the resistance always returns to the initial state after release. Figure 5c,d shows the ΔR/R_0_ changes during 500–520 and 7500–7520 stretch-release cycles. After 8000 cycles, the ΔR/R_0_ only has a small increase, and the sensor remains stable. The channel structure of the sensor provides good interfacial stability for MWCNT crack stretching. Since the MWCNT is deposited inside the PDMS channel and forms a three-sided contact with PDMS, compared with other 2D MWCNT deposited on the substrate surface, the MWCNT in this paper forms a 3D structure, which ensures good interfacial contact. Meanwhile, the crack is fully extended and no generated new cracks after a certain tensile strain allowing the sensor resistance change to be stable in the 0–4200% interval.

### 3.2. Applications of the Transparent Strain Sensor with the Embedded MWCNT Network

The flexible strain sensors based on PDMS prepolymer and cross-linker ratio of 15:1 show high sensitivity and transparency. The optical transparency of the sensor reduces any inconvenience to the user while wearing it during normal activity. This increases the acceptance of wearable devices, allowing the sensor to be more useful in terms of applications. The strain sensor with the embedded MWCNT network could be potentially useful in wearable devices, biomedicine, and health monitoring.

**Figure 6:**
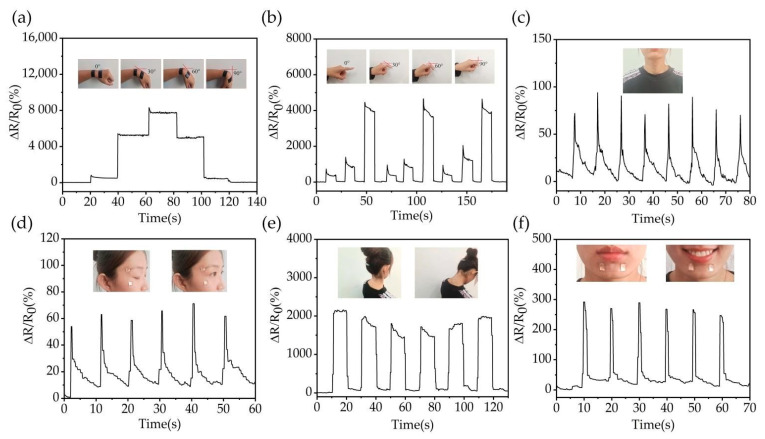
Applications of the strain sensor with the embedded MWCNT network in monitoring various human motions. Change of ΔR/R_0_ for: (**a**) bending of wrist, (**b**) bending of finger, (**c**) swallowing of food, (**d**) blinking, (**e**) bending of neck, and (**f**) smiling.

To evaluate the possibility of sensor applications in wearable devices, tests were conducted on finger or wrist flexion, swallowing movements, changes in eye movements and facial expressions, and changes in neck flexion (Figure 6). To isolate the human body resistance from the measurement results, medical tape or insulating tape was used to adhere the strain transducer to the human skin surface. When the sensor is attached to the wrist and finger (Figure 6a,b), it can clearly detect the changes in resistance when the wrist and finger are bent. The sensor identifies different resistance changes; the normalized resistance value of the wrist reaches 8000% and that of the finger is 4000%. Meanwhile, subtle human motion such as swallowing and eye movements can also be promptly and accurately monitored by the strain sensor (Figure 6c,d). The ΔR/R_0_ responses reached 70% or more with human swallowing.

Due to the development of technology, daily life is inseparable from high-tech products such as mobile phones and computers. “Phubbing” has become synonymous with people, and prolonged bending of the neck is dangerous to human health. The sensor can monitor the neck posture: the resistance tends to be stable when the neck is upright, but ΔR/R_0_ increases to 2000% when the neck is bent (Figure 6e). Moreover, the sensor can quickly respond to signal changes. Due to their high sensitivity and fast response times, the proposed flexible strain sensor with an embedded MWCNT network can improve “Phubbing”. Furthermore, the sensor can also be used to monitor changes in human facial expressions by mounting the sensor on the chin (Figure 6f). When smiling, the muscles in the chin stretch to generate strain in the sensor, thus resulting in a change in the normalized resistance values. Human facial activity can be determined by analyzing the change in resistance values. These results obviously indicate that the strain sensor with the embedded MWCNT network has good responsiveness, high sensitivity, and high transparency. In addition, the sensor can also be applied to small and medium deformation tests and has the potential for use in real-time medical treatments.

## 4. Conclusions

In summary, we have successfully fabricated a transparent strain sensor with an embedded MWCNT network to monitor human movement. The embedded PDMS channel film was fabricated via an EFD microscale 3D printing and molding hybrid process free of time-consuming procedures and material wastage. Meanwhile, the PDMS channel film has the advantage of high degree of freedom, and is suitable for filling other types of conductive materials, and the performance of the sensor can also be improved by designing different structures. The sensor was based on the cracking mechanism and exhibited excellent characteristics in terms of sensitivity, transparency, stability, and response time. The sensing performance of the flexible strain sensor was systematically characterized, and the sensitivity of the sensor could be improved by changing the ratio of PDMS prepolymer and cross-linker (10:1, 15:1, and 20:1). The detection range could thus be reduced. Meanwhile, the PDMS channel network provides good protection for MWCNTs. The resulting sensor shows high sensitivity (the GF of 90 at a strain of 6.6%, the GF of 285 at a strain of 14%, the GF of 1500 at a strain of 20%), stability (8000 cycles), responsiveness, and optical transparency (>84%). These features make the sensor promising for various human motion–detection applications. Furthermore, the manufacturing of sensors using EFD 3D printing and molding hybrid process offers high efficiency and low cost, which are suitable for mass production. The proposed sensor manufacturing method offers a new technological route for the application of 3D printing technologies in wearable electronic devices.

## Figures and Tables

**Table 1:** Summary of performance results of carbon-based strain sensors recently reported.

References	Materials	Strain Range	GF	Mechanical Stability	Transmittance
This work	MWCNTs/PDMS	20%	90 (6.6%) 285 (14%) 1500 (20%)	8000 cycles at 14% strain	84%
[2]	SWCNTs/CB/PDMS	300%	0.91 (0–100%) 3.25 (100–255%) 13.1 (255–300%)	2500 cycles at 200% strain	N/A
[9]	CNTs/Ecoflex	20%	14.5 (0–20%)	50 cycles at 20% strain	N/A
[10]	SWCNTs/PDMS	280%	0.82 (0–40%) 0.06 (60–200%)	10,000 cycles at 200% strain	N/A
[12]	CNTs/Graphene/PDMS	120%	182.5 (0–3%) 45.6 (3–57%) 70.2 (57–90%) 186.5 (90–120%)	10,000 cycles at 40% strain	N/A
[28]	CNTs/PDMS	30%	3 (0–10%) 4 (10–20%) 6.6 (20–30%)	1000 cycles at 40% Strain	92%
[48]	MWCNTs/PDMS	10%	17.6 (0–5%) 134.1 (5–8.75%) 330 (10%)	2000 cycles at 7% strain	87%

## Data Availability

Data sharing is not applicable to this article.

## Supplementary Materials

The following are available online at https://www.mdpi.com/article/10.3390/nano12010120/s1, Figure S1: TGDSC image of MWCNTs, Figure S2: SEM of the dispersion of MWCNTs unfilled in PDMS channel and the dispersion of MWCNTs filled PDMS channel.
