# Supplementary material for: Cost-Effective Fabrication of Transparent Strain Sensors via Micro-Scale 3D Printing and Imprinting
Source: Nanomaterials (Basel). 2021 Dec 30;12(1):120. doi: 10.3390/nano12010120 (PMC8746503; doi:10.3390/nano12010120)
Supplement: Supplementary file 1 [file nanomaterials-12-00120-s001.zip › nanomaterials-1508394-supplementary.pdf]

nanomaterials

Supplementary Materials

Cost-Effective Fabrication of Transparent Strain Sensors via Micro-Scale 3D Printing and Imprinting

Rui Wang [1,2], Xiaoyang Zhu [1,2,*], Luanfa Sun [1,2], Shuai Shang [1,2], Hongke Li [1,2], Wensong Ge [1,2] and Hongbo Lan [1,2,*]

1 Key Lab of Industrial Fluid Energy Conservation and Pollution Control, Ministry of Education, Qingdao 266520, China; wr1970627@163.com (R.W.); sunluanfa@163.com (L.S.); shangshuai0602@163.com (S.S.); LHK1164072308@163.com (H.L.); g17864208754@126.com (W.G.)
2 Shandong Engineering Research Center for Additive Manufacturing, Qingdao University of Technology, Qingdao 266520, China
* Correspondence: zhuxiaoyang@qtech.edu.cn (X.Z.); hblan99@126.com (H.L.)

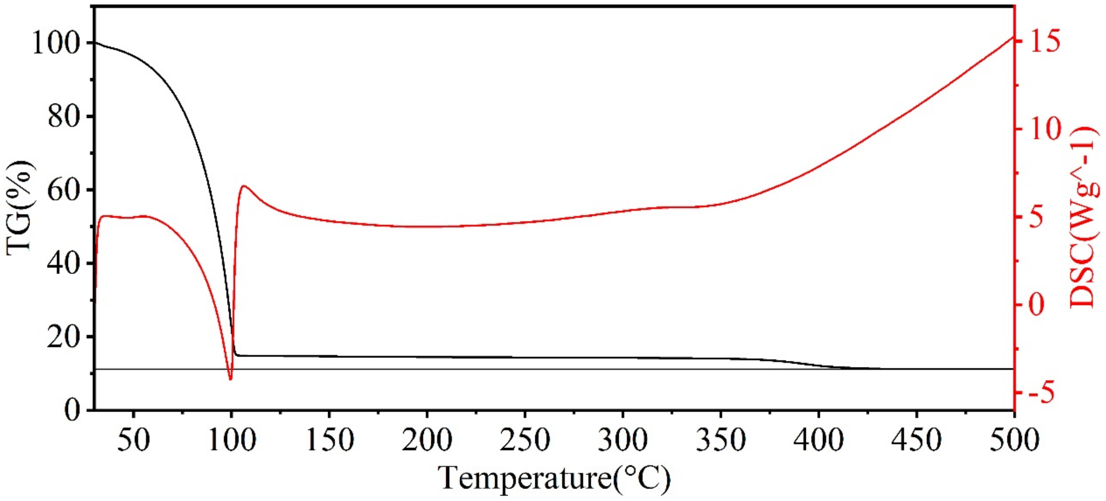

Figure S1. TGDSC image of MWCNTs

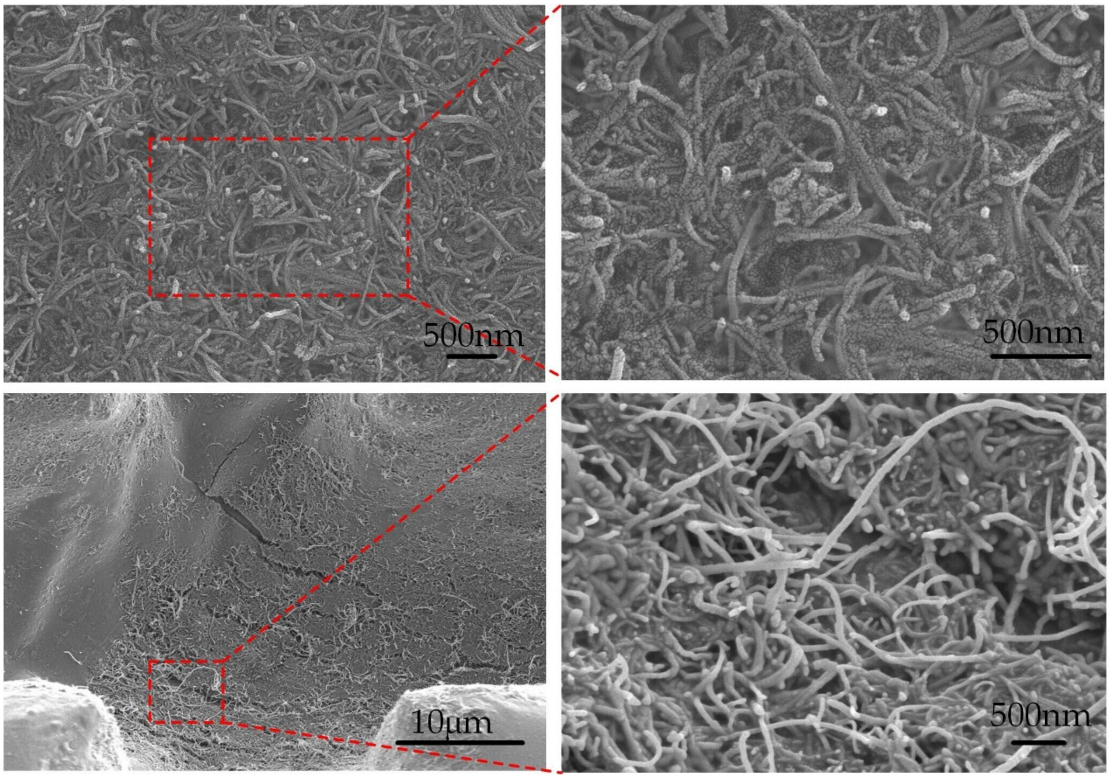

Figure S2. SEM of the dispersion of MWCNTs unfilled in PDMS channel and the dispersion of MWCNTs filled PDMS channel.
